# Informed consent for HIV cure research in South Africa: issues to consider

**DOI:** 10.1186/1472-6939-16-3

**Published:** 2015-01-15

**Authors:** Ciara Staunton

**Affiliations:** Centre for Medical Ethics and Law, Department of Medicine, Faculty of Medicine and Health Sciences, Stellenbosch University, PO Box 19063, Francie van Zijl Drive, 7505 Tygerberg, South Africa

**Keywords:** HIV/AIDS, HIV/AIDS cure, Informed consent, Therapeutic misconception

## Abstract

**Background:**

South Africa has made great progress in the development of HIV/AIDS testing, treatment and prevention campaigns. Yet, it is clear that prevention and treatment campaigns alone are not enough to bring this epidemic under control.

**Discussion:**

News that the “Berlin patient” and the “Mississippi baby” have both been “cured” of HIV brought hope to people living with HIV/AIDS in South Africa that a cure for HIV/AIDS is within reach. Despite the recent setbacks announced in the “Mississippi Baby” case, protocols aimed at curing HIV/AIDS are being developed in South Africa. However with evidence to suggest that participants in clinical trials do not understand the basic concepts in the informed consent process, there is concern that future participants in HIV/AIDS cure research will lack comprehension of the basic elements of future clinical trials that aims to cure HIV/AIDS and confuse research with clinical care.

**Summary:**

Research ethics committees have an important role to play in ensuring that participants understand the basic concepts discussed in the informed consent process, that they understand that research is not clinical care and they are unlikely to benefit from any early phase trials seeking to cure HIV/AIDS.

## Background

Since the days of AIDS denialism [1–3], South Africa’s policy on HIV/AIDS has seen a transformation with the introduction of national treatment, testing and prevention campaigns [4]. Despite an increase of 1.2 million new infections since 2008, the rate is stabilising and access to treatment is improving [5]. By mid-2012 31.2% of people living with HIV (PLHIV) were on treatment, up from 16.6% in 2008 [5] and South Africa has now passed the “tipping point” whereby more people are accessing treatment than there are new infections [6]. This has come on foot of a successful testing campaign which has seen rates of HIV/AIDS testing increase [7]. However with 6.4 million people (12.2% of the population) living with HIV/AIDS [5], South Africa has the highest number of HIV/AIDS infected individuals worldwide and HIV/AIDS continues to be a considerable challenge facing the country [8].

Yet life is improving for PLHIV. The availability of anti-retroviral therapy (ART) has helped increase life expectancy from 49.2 years prior to the availability of ART to 60.5 years by 2011 [9]. PLHIV in South Africa can now also expect to have 80% of normal life expectancy provided they start treatment before their CD4 count drops below 200 [10]. Yet this increased provision of treatment has a significant economic impact on the nation. Although a recent renegotiation of a fixed dose ARV resulted in a 40% price reduction, South Africa’s programme is the largest treatment programme in the world and a considerable economic burden for the state [11]. In August 2012 the US government announced that it would cut the United States President’s Emergency Plan for AIDS Relief (PEPFAR) budget for South Africa in half by 2017, at which point South Africa will have to cover the full cost of its HIV/AIDS programme. The process of the government taking full responsibility for the programme has led to the closing of HIV/AIDS treatment centres created by PEPFAR and patients’ moving to government run centres, centres which regularly face long waiting times and medication shortages [12]. Equally problematic is the high prevalence rate [8]. Thus, while treatment and prevention campaigns can reduce infection rates and improve the quality of life for PLHIV, treatment and prevention campaigns are not enough to bring the epidemic in South Africa under control.

News that the “Berlin patient” [13] and the “Mississippi baby” [14] have both been “cured” [15, 16] of HIV likely brought hope to PLHIV in South Africa that a cure for HIV/AIDS is within reach, a hope which may be fuelled by further announcements of other babies who have also been cured [17]. Although these early reports of HIV/AIDS cures have been somewhat dampened by the reports of the return of HIV in the “Mississippi Baby” [18] and the “Boston patients” [19], it appears that the search for a cure is gaining momentum. Yet these new developments bring a fresh set of ethical challenges that must be addressed prior to the commencement of any trial aimed at curing HIV/AIDS in South Africa. Of particular concern is that at some point during such a clinical trial, participants who are doing well on their ART may have to go off their treatment and try some new, experimental intervention [20]. Participants will be asked to assume the documented risks of treatment interruption [21], despite the very low possibility of personally benefiting in any early phase trial. The recent setbacks of these early cases of cure illustrate that there is a lot that is still unknown about HIV/AIDS cure research and there is the real possibility that HIV may return in any early phase trial.

With such unknowns, it is critical that the conduct of the trial meets the highest ethical standards. Concerns about trial design and the potential withdrawal of ARVs must be discussed and addressed. Equally important is a robust informed consent process that informs participants about these risks. However with growing evidence in South Africa that trial participants do not understand the information provided during the consent process and have unrealistic expectations of possible personal benefit, questions must be asked about the current consent process [22–25]. This paper will examine the informed consent process in South Africa and consider whether additional safeguards must be put in place to protect participants in future HIV/AIDS cure research. In particular it will discuss the experiences of the informed consent process during clinical trials in South Africa and the potential implications they may have on any future trial aimed at finding a cure for HIV/AIDS in South Africa. Good ethical oversight is imperative in this process and the role that RECs should play in this oversight will be discussed.

## Discussion

### Informed consent in South Africa: the legal landscape

Informed consent is a fundamental ethical principle that was first codified in the Nuremberg Code in the wake of the Nazi experiments [26, 27]. It has since been replicated in all major ethical codes including the International Covenant on Civil and Political Rights, the Declaration of Helsinki and the Council for International Organisations of Medical Sciences (CIOMS) guidelines. In South Africa, the principle is enshrined in both the Constitution and the National Health Act 2003, highlighting its importance. The national research ethics guidelines *Ethics in Health Research: Principles, Structures and Processes* require that all participants must be informed of the risks and benefits, purpose of the research and the procedures to which they will be subjected to. These must be understood and consent must be voluntary and based on this information. The guidelines are cognisant of the fact that due to access to health care and their education levels, many people involved in research in South Africa are considered to be vulnerable and researchers must be mindful of this vulnerability [28]. The clinical trial guidelines further detail what should be included in the informed consent form (ICF). For all clinical trials, the original signed consent form must be kept with the trial records with one copy stored in the patients’ records and a second copy given to the participant to take home [29]. These guidelines have also been supplemented with specific guidance on informed consent in *HIV Preventive Vaccine Research* guidelines [30], and other international guidelines on HIV/AIDS research, such as the UNAIDS documents, may also be followed in clinical trials [31]. Together, these guidelines regulate the informed consent process in South Africa and detail the procedure and protections which must apply to all HIV/AIDS clinical trials.

### Experiences with informed consent in South Africa

Despite ample guidelines on the matter, obtaining informed consent in clinical trials is problematic in South Africa. This challenge can be partly attributed to the dual purpose of the ICF: the institution or principal investigator (PI) use it as a tool to indemnify them from litigation as a result of the increasingly litigious culture emerging in Western societies [32], while the ethical guidelines focus on informing the participants of all the risks associated with the research. Thus from a document which has its origins in promoting ethical research, it has evolved to become an increasingly legalistic document which can be difficult to read and decipher its contents [33]. This is challenging as a document which is primarily designed to offer legal protection will be markedly different to a document aimed at protecting trial participants through enabling them to make an autonomous choice. However the South African guidelines do require that research must be explained in “a clear, simple and culturally appropriate manner” thus one could assume that effort is given towards establishing the comprehension of the ICF. Yet there is growing evidence that certain key requirements of informed consent are not met in clinical trials in South Africa and basic understanding is lacking [22–25, 34], including during HIV vaccine trials [35, 36].

The belief that consent is not voluntary or is somehow linked to care is emerging as a common misconception in four reported studies. In one antenatal clinic, it was found that 93% of participants thought that they could not withdraw from the study and 28% thought that they would not receive care if they did not participate in the study [22]. These findings were replicated in a similar study in Bloemfontein where 24.2% thought they could not withdraw at any time and 92.3% believed that they would not receive good medical care if they did withdraw from the study [23]. Recently it was shown that half of those surveyed felt that they did not have a choice about getting tested for HIV/AIDS, with many believing that access to healthcare was contingent on their consent to the test [25].

This lack of understanding also extends to key aspects of the trial itself. Participants in an influenza trial underwent a thorough informed consent procedure that involved a community meeting, an individual discussion with a study nurse and a discussion with the study doctor on the day of the trial. Despite these repeated consultations, participants did not understand concepts such as randomisation or placebo and 38% of participants stated that they would not have continued with the study if they had known that they would receive an inactive placebo [24]. Similarly, the Bloemfontein study found that although participants had at least 8 years of education, they lacked a basic understanding of the clinical trial and their consent could not be considered to be informed [23].

Clearly comprehension of the often complex information is problematic and there is evidence to suggest that the ICFs are not understandable by the general population. The readability of the forms have been found to be above the education level of the general population [34]. South Africa is ranked 146^th^ out of 148 countries for education in light of its low primary and tertiary enrolment rates [37] and functional illiteracy is at 16.5% [38]. There is therefore likely to be a great imbalance in knowledge between the researcher and the participant [39], but the consent process does not appear to address this imbalance and the basic concepts of the trials do not appear to be appropriately communicated or understood.

With such challenges facing understanding of the trials, it is unsurprising that it has been indicated that therapeutic misconception (TM) may be a problem in South Africa [24], a trend which has been seen elsewhere [40–42]. TM occurs when research participants assume that decisions will be made based on their best clinical interest [43]. In other words, research is confused with clinical care. This is distinct from the therapeutic misestimation where the participant may underestimate the risk or overestimate the benefit [44], and therapeutic optimism which occurs when participants are unduly optimistic about the potential success of the research [40]. What distinguishes TM is that participants fail to “grasp that the risks they face from participating in research protocols are inherently different from those involved in receiving ordinary treatment” [43]. They fail to appreciate that decisions will be made based on the best interest of the trial. Thus the participant cannot be said to be making a free, fully informed decision.

There is very little empirical data on TM and motivations to participate in a trial in South Africa, but a recent study analysing motivations for enrolling on a Phase 3 oncology study has suggested that participants do expect benefit from participating in a trial. The participants were quite optimistic about the outcome of the trial and this is not something which should be discouraged, but they had a poor understanding of phase 3 research and believed that the clinical trial posed very little risk to them [36]. These findings indicate that participants did not understand the information provided during the informed consent process and suggest that TM may also be an issue in South Africa.

### Informed consent and HIV/AIDS cure research

In an attempt to protect them from legal liability, disclosure of information by the research team is unlikely to be a problem in South Africa. Rather the challenges lie in comprehending this information. Researchers are not absolved of their duty during the consent process by simply providing the participants with information. The ethical guidelines task the PIs with ensuring that participants understand the information provided, but must all information be understood and what are the implications for future HIV/AIDS cure clinical trials if they are not satisfied?

It has been proposed that the rationale for the study, technical issues, technical consequences, methodological issues, practical aspects involved in personal participation, the costs and benefits of participation in the study and the personal implications of participation in the study should be at least understood before involvement in a HIV vaccine trial [33]. These certainly should also be disclosed for HIV/AIDS clinical trials, with a strong focus on the risks of the trial as there are likely to be very real risks in early phase HIV/AIDS cure research. For HIV positive individuals who are otherwise healthy and on highly active ART with an undetectable viral load who take part in a cure trial, it is possible that they will be asked to stop taking their treatment [20, 45]. All but one of purported cases of a “cure” for HIV has seen the disease return demonstrating the real risk that HIV may return. Furthermore in two Boston patients who underwent treatment similar to the Berlin patient, HIV/AIDS returned in both patients resulting in one becoming resistant to their treatment [46]. In addition to these documented risks is the possibility of unknown side effects associated with the treatment. Current evidence suggests that there may be real challenges in effectively communicating key concepts of the trial to a study group that will be largely drawn from a poorly educated population. Thus can we realistically expect participants to be able to sufficiently understand the very real risks and benefits of enrolling in an early phase HIV/AIDS cure trial?

In addition is the real concern of TM. Africa has a history of people willing to try unproven “cures” in the hope of being free from HIV, thus demonstrating that people may be willing to assume great risks in the hope of a cure [47, 48]. The pursuit of this hope may lead them to enrol in an early phase HIV/AIDS cure trial despite being told that there will unlikely be any individual benefit for them. TM is a real problem in early phase research as unlike in phase 3 or 4 studies, there is unlikely to be any direct medical benefit and the research can be in no way linked to a participants’ clinical care. In light of the *Malan* study and evidence that PLHIV will try unproven treatments in their search for a cure, the research team must guard against TM in HIV/AIDS cure research. The simple use of the word “cure” as one of the study aims may be enough to ignite the possibility of TM. Yet participants cannot be misled; if it is hoped that the clinical trial will find a cure for HIV, this should be stated in the patient information leaflet and ICF, but care must be taken around contextualising the research to participants. The challenge for the research team is if after a thorough informed consent process the research team suspects that the participant is simply enrolling because of the remote possibility of a cure, should they be refused to join the trial?

The South African guidelines focuses on understanding only and do not directly address this point. Horn and Grady describe understanding as “having an accurate grasp of the available options and the consequences of choosing one over the other”. If a participant understands the risks and benefits and the implications for their enrolling in a study, but due to some irrational hope that they may be cured they still enrol, technically they satisfy the requirements of informed consent. The risks are understood but are disregarded in the hope of a possible cure. However is their desire for a cure affecting a participant’s voluntariness? Or have they simply decided that the risks are worth taking, despite the low probability of success?

Horn and Grady argue that TM cannot be tolerated as it “fundamentally misrepresents the choice of research participants”. Clearly an ability to understand the difference between clinical care and research is essential and would fall under the requirement of understanding under the South African guidelines. Participants who do not understand the difference between research and clinical care do not understand the basic fundamentals of research and thus cannot give free, informed consent. Yet it is less clear for the overly optimistic participant who has decided that the risks are worth taking despite the low probability of success. On this point, Horn and Grady consider that the therapeutic misestimation, whereby the participant may underestimate the risk or overestimate the benefit, can be tolerated when the benefit or risk is not large [44]. Turning back to the requirement of understanding in the South African guidelines, the therapeutic misestimation is arguably a misunderstanding as they fail to appreciate the degree of risk or benefit, but it may also be a symptom of being unduly optimistic. They may fully understand the risks and the low probability of benefit, but continue to hold onto their belief. Is undue optimism something we should guard against particularly when their optimism is unlikely to materialise in early phase trials or is it enough that they understand the risks and benefits?

### The role of RECs in cure research

Clearly trusting the integrity of the research team to make an ethical decision is necessary. They are to conduct the trial in accordance with the protocol submitted to the REC and South African ethical guidance and ensure that the elements of informed consent have been met. The guidelines focus on the need for disclosure and comprehension but offer little practical guidance on how this is to be achieved. The onus is indeed on the PI to ensure that the informed consent process meet these criteria. The degree of therapeutic misestimation that is acceptable before it conflicts with the requirement of comprehension is not one that can be succulently stated in any guidance. Rather it is a decision that must be made by the research team. Equally, they must determine if the participants sufficiently understands the information provided for the consent to be valid. There is also a role for the RECs in overseeing the ethical conduct of future HIV/AIDS cure trials. They can provide a number of gate keeping functions, including a role in promoting the comprehension of participants whilst guarding against TM.

First they must be assured that information is not simply conveyed to participants, but there is a real effort to ensure participants understand the risks involved in the research as well as the basic principle of informed consent. Simplified ICFs do improve understanding [49], but they alone may not be enough. The *Coletti* study evaluated a prototype informed consent process for HIV vaccine trials and it found that there was a substantial increase in knowledge and understanding of the trial [50]. A similar consent form could be developed for cure research. Part of this could also include the development of an educational tool that can visually convey certain aspects of the research. This should be in addition to the ICF and the patient information leaflet. Although the impact of these tools will need to be assessed, they may go some way towards improving the comprehension of participants in clinical trials. A trial may aim to achieve a functional cure that controls the symptoms without eradicating the virus and a trial which is aimed at finding a sterilising cure that leads to the complete removal of HIV from the body. These are complex concepts that are not easily described or understood, in particular how the virus may remain in the body but they will no longer need to take treatment. As the influenza study has demonstrated that repeated consultations do not necessarily lead to improved comprehension, the REC should be satisfied that the research team has a plan in place to ensure the comprehension of participants that goes beyond the patient information leaflet and the ICF.

Secondly RECs must be cognisant of the risk of TM in HIV/AIDS cure trials and ensure that the information presented during the informed consent process should not raise any undue expectations. While care must be taken that the ICF clearly explains the risks of the research, explaining the research may be challenging. It has been suggested that “sterilising cure”, “functional cure” or “sustained virological response (SVR)” could be used other than “cure”. Such concepts however are unlikely to resonate with participants. They are unlikely to understand the distinction between functional and sterilising cure and SVR may not be easily explained. “Remission” however, is a term that is commonly used in the cancer context and does not evoke the same hope or expectation as “cure”. It adoption in this context has been recommended as one way in which the TM may be kept in check [51]. It is not only more familiar to participants, but it reflects the uncertainty in future outcomes [16].

Finally although these measures may be of benefit in the informed consent process, understanding of key elements of the trial can still be lacking [49]. Trial counsellors have reported that at times they believe that participants do not understand the information provided, despite their claims to the contrary [52]. An educational tool can improve comprehension, but it in itself is not enough to satisfy that the information is understood. Lo and Grady suggest that due to the potential distorting effect that the desire for a cure may have, understanding of the potential risks and benefits should be assessed [45]. RECs can require such an assessment of understanding, in much the same way as they require the ICFs to be understandable. Such assessments are not new and were used during HIV Prevention Trials Network studies where participants were given two opportunities to demonstrate comprehension [53]. The development of these assessments are complicated as reported levels of understanding can be affected by who asks the question, the formality of the setting, the phrasing of the question [54], as well as the method of assessment [55]. However it is crucial that efforts are made towards the development of a tool for cure trials to ensure that participants understand the basic concepts of their involvement in a clinical trial, as well as the benefits and risks involved in such a trial. Assurance of understanding is essential. By requiring these tools, RECs can put added protection into the system by querying with PIs how they intend to assess understanding and require justification if no method is put forward.

Unfortunately such a tool will likely only indicate instances of therapeutic misconception but may not identify cases of therapeutic misestimation or irrational hope. Identification of such instances is likely to arise during more informal methods of assessment that are often adopted such as the reading of a participant’s body language. In any case the South African guidelines are silent on whether unduly optimistic participants should be precluded from enrolling, requiring only that the information is understood. This will ultimately be a judgment call to be made by the PI in consultation with other members of the research team and in light of the potential risks that is underestimated or the likely benefits that is overestimated.

### Introducing cure trials in South Africa: the way forward

Currently there are no clinical trials ongoing in South Africa aimed at curing HIV/AIDS but with protocols under development, the time is now for all stakeholders to consider these issues and develop these tools and guidelines for such clinical trials. Once the issues which may affect understanding or motivate participants to enroll on such a trial are known, stakeholders can put in place additional safeguards to ensure that the informed consent process for clinical trials that aim to cure HIV/AIDS is robust in South Africa.

It is clear that the informed consent process in South Africa requires further research and analysis as the limited studies on this issue do show that there are flaws in the process. The process is complicated by the need to explain complex scientific issues to participants who often have low levels of education. Thus the problems in the informed consent system must be identified and addressed. It is important that the informed consent process is based “on empirically valid and reliable methods that serve the goals of informed consent, rather than on educated guesses about what and how to inform prospective subjects” [56]. In the development of HIV/AIDS cure protocols, these challenges must be kept in mind and every effort must be made to bolster the informed consent process and protect participants. In particular, there is a need for a discussion on comprehension of the information and what must be understood before the consent can be deemed informed. It is possible that participants will understand the basics of the trial but misestimate the risk-benefit ratio. Is it ethical to permit such participants to enroll on an early-phase trial?

This is not a call for new or additional guidance. Current guidelines addresses issues such as community participation, involvement of vulnerable populations, children and women as well informed consent and monitoring informed consent [31]. They do require that the investigator must be assured that the participant understands the information and methods to evaluate understanding are encouraged [31]. However in light of the considerable risks of treatment interruption and the relatively healthy pool that the participants are likely to come from, there is a need for a discussion to inform both REC members and researchers on the informed consent process and the safeguards which must be put in place to address concerns about understanding and TM. What must be understood is likely to vary according to the trial design and the intervention used, but a general discussion on the understanding of treatment interruption as well as whether therapeutic misestimation is permissible is needed. This will assist the research team in determining whether the consent is informed and also help RECs when they evaluate the information in the patient information leaflet, the ICF and any educational tool or assessment of understanding.

## Summary

Moving forward, a discussion is necessary on the essential elements that should be understood during the informed consent process in South Africa. Requiring complete understanding of all the procedures involved in a HIV/AIDS cure trial will likely result in no participant meeting the consent criteria. A degree of misunderstanding and confusion of these complex scientific concepts is to be expected and must be acceptable. However some agreement is necessary on the degree of misunderstanding that is acceptable and on what particular issues.

Research is currently underway at Stellenbosch University in Cape Town to investigate the views of participants on some of the issues raised here. In collaboration with the University of North Carolina, Chapel Hill and the University of Carolina, Guangzhou, this project aims to consider the social and ethical aspects of HIV Cure research. Findings from this study can inform these discussions in South Africa and also inform research teams in developing their protocols and RECs in reviewing these submissions. In the interim, RECs should discuss the possibility of some of the measures discussed here with the PIs in any trial aimed at curing HIV/AIDS to increase understanding and bolster the informed consent process.
